# Work outcome in yet undiagnosed patients with non-radiographic axial spondyloarthritis and ankylosing spondylitis; results of a cross-sectional study among patients with chronic low back pain

**DOI:** 10.1186/s13075-017-1333-x

**Published:** 2017-06-17

**Authors:** Lonneke van Hoeven, Annelies E. R. C. H. Boonen, Johanna M. W. Hazes, Angelique E. A. M. Weel

**Affiliations:** 1 Department of Rheumatology, Erasmus MC, Wytemaweg 80, 3015 CN Rotterdam, The Netherlands; 2 Department of Rheumatology, Maasstad Hospital, Maasstadweg 21, 3079 DZ Rotterdam, The Netherlands; 3 Department of Rheumatology, Maastricht University Medical Centre, P. Debyelaan 25, 6229 HX Maastricht, The Netherlands

**Keywords:** Axial spondyloarthritis, Low back pain, Work participation, Patient reported outcome measures, Burden of disease

## Abstract

**Background:**

To understand the impact of yet undiagnosed non-radiographic axial spondyloarthritis (nr-axSpA) and ankylosing spondylitis (AS) on work outcomes in a cohort of patients with long-lasting chronic low back pain (CLBP).

**Methods:**

Data were used from a primary care CLBP cohort that was established to understand the prevalence of nr-axSpA and AS. Clinical characteristics comprised measures of back pain (visual analogue scale), inflammation (C-reactive protein) and physical functioning (Roland Morris Disability Questionnaire (RMDQ)). Worker outcomes comprised a question on employment and the Work Productivity and Activity Impairment (WPAI) questionnaire, distinguishing absenteeism, presenteeism, and overall work impairment in those employed and activity impairment in all patients. For each disease subgroup, employment ratio compared to the general population was assessed by indirect standardization. Factors associated with work productivity were explored by zero inflated negative binomial (ZINB) regression models.

**Results:**

Patients with CLBP (*n* = 579) were included (41% male, mean age 36 years), of whom 71 (12%) were identified as having nr-axSpA and 24 (4%) as having AS. The standardized employment ratios were 0.89 (95% CI 0.84–0.94), 0.97 (95% CI 0.85–1.09) and 0.81 (95% CI 0.56–1.06) for patients with CLBP, nr-axSpA and AS, respectively. Scores for the WPAI subdomains were not significantly different between patients with CLBP, nr-axSpA or AS. The ZINB models showed significant associations between visual analog scale (VAS) score for pain and RMDQ and work productivity.

**Conclusion:**

The impact of yet undiagnosed nr-axSpA and AS on patients’ work outcomes was substantial but was not significantly different from those of patients with long-standing CLBP. Variables significantly associated with reduced work productivity were VAS for pain and RMDQ score.

**Electronic supplementary material:**

The online version of this article (doi:10.1186/s13075-017-1333-x) contains supplementary material, which is available to authorized users.

## Background

Low back pain (LBP) is a major health and societal problem affecting more than 80% of adults at some point in their lives [1]. Between 10 and 28% of episodes of LBP persist for more than 12 weeks and become chronic complaints [2]. A study has shown that up to 24% of chronic LBP (CLBP) in young adults can be explained by axial spondyloarthritis (axSpA) [3]. AxSpA is an auto-inflammatory disease of the spine that is potentially treatable. Two subtypes of axSpA can be distinguished; in non-radiographic axSpA (nr-axSpA) either sacroiliitis is visible on magnetic resonance imaging (MRI) or human leukocyte antigen B27 (HLA-B27) is positive, and in addition one or two so-called SpA features are present [4]. In those with radiographic axSpA, structural changes are visible on the X-ray of the sacroiliac joints and this subtype corresponds to what is commonly known as ankylosing spondylitis (AS).

Although the new classification criteria for axSpA were developed to enhance early recognition and subsequently provide earlier and better treatment, the profile of those in whom the diagnosis of axSpA is wrongly missed is not completely elucidated. This is important, as it could provide insight into the reversible burden of the disease when diagnosis would have made earlier. Several studies report an overall comparable clinical burden in patients with AS and nr-axSpA [5, 6], however these patients were referred in prospective settings and not wrongly missed.

The clinical burden of a chronic inflammatory disease can be expressed in terms of disease activity and impaired function, but also in work participation [7]. The impact of undiagnosed axSpA on the patients’ capacity to work is important from the perspective of the patient and their families [8], and from the societal perspective when calculating indirect costs to determine the economic burden of a disease and the possible return on investment by using case-finding strategies for axSpA patients. Moreover, such data can help us understand the level of support patients with axSpA might need to help them to remain active in the labor force and safeguard their career perspective.

Some data are already available on the impact of AS and axSpA on work participation. A review of work outcomes in AS indicates that patients with longstanding disease incur official work disability up to three times more frequently, and that there had already been substantial work loss at the time of diagnosis [9]. Also a recent study in patients with early axSpA reported that within only 5 years of diagnosis, 19% of patients with axSpA were not employed because of the axSpA [10]. Also, in those still working, 28% and 48% of the patients reported having sick leave and reduced productivity at work [10]. A recent medication trial in patients with nr-axSpA showed an improvement in worker productivity of 9.6 h/week in the patients with nr-axSpA who had a good response to their treatment (assuming a 40-hour working week) [11], suggesting that early recognition of axSpA might prevent adverse work outcomes. It is already known that CLBP has a significant impact not only on work productivity, but also on daily activities [12, 13].

The aim of this study was to investigate work outcomes in yet undiagnosed patients with nr-axSpA and AS among a cohort of patients with CLBP. The specific aims were to compare the employment of patients with AS, nr-axSpA and CLBP with the general population, to explore whether these diagnostic groups differed in sick leave and productivity at work and to explore which demographic and disease characteristics contributed to sick leave and productivity at work.

## Methods

### Study population

All patients from the second cross-sectional Case Finding Axial SpondyloArthritis (CaFaSpA 2) study were included [14]. The study was performed in 2011 and 2012 in the south-western part of the Netherlands. Ethics approval from the Medical Ethical Committee from the St. Elisabeth Hospital in Tilburg, the Netherlands was received. Written informed consent was obtained from all participants at the research center, before any assessment was performed.

Patients with CLBP ages 18-45 years were selected by ICPC code L03 (nonspecific low back pain) from general practice (GP) records and invited to participate if the CLBP had been present for at least 3 months. Participating patients were examined by a rheumatologist or an experienced research nurse, i.e. recording of medical history and physical examination, including identification of features of SpA. All assessments and definitions adhered to the descriptions in the Assessment of SpondyloArthritis International Society (ASAS) handbook [15]. Blood was drawn to determine HLA-B27 positivity, C-reactive protein (CRP) (normal range 1–10 mg/L) and erythrocyte sedimentation rate (ESR) (normal range 0–15 mm Hg/min). X-ray and MRI of the sacroiliac joints were obtained in all patients. A definitive diagnosis of sacroiliitis was made according to the ASAS MRI criteria [4] or the modified New York criteria for the X-ray [16], by one of three trained radiologists, who were blinded to the clinical outcomes, laboratory data and results of other imaging methods. The primary outcome of this study was to identify new patients with axSpA using the ASAS classification criteria [4]. None of the newly identified patients with axSpA had received any treatment with non-steroidal anti-inflammatory drugs (NSAIDs) or biologic agents.

### Questionnaires

To assess disease severity patients completed the Bath AS Disease Activity Index (BASDAI) [17], Ankylosing Spondylitis Disease Activity Score (ASDAS-CRP) [17], a visual analog scale (VAS) for pain (range 0–10) and the Roland Morris Disability Questionnaire (RMDQ) [18]. The RMDQ was developed to measure limitations in physical functioning in patients with CLBP. It consists of questions about impairment and limitations in different activities due LBP. Patients indicate if a question is applicable to them (score = 1) or not (score = 0). The score can range from 0 to 24 and a higher score indicates a higher level of disability.

### Socioeconomic status and worker productivity

All participants completed questions about their highest achieved educational level; low (elementary school), medium (high school) and high (university), current work status (employed, or not employed), and the number of working days and working hours per week in those employed. To assess whether a patient was work-disabled or not work-disabled, we asked the patient is there was an official disapproval of the insurance company doctors. Answers to an open question about occupation were classified into non-manual (administrative, scientific and managerial professions) and manual (industrial, commercial, servicing, transportation and agricultural) jobs, using the International Standard Classification of Occupation (ISCO)-08 major groups [19].

Finally, the Work Productivity and Activity Impairment (WPAI) questionnaire was completed, which evaluates four subdomains; absenteeism, presenteeism, work impairment and activity impairment, due to back problems in the past 7 days [20]. The subdomains are all expressed in percentages; absenteeism (percentage work time lost), presenteeism (percentage productivity loss at work), work impairment (absenteeism and presenteeism combined) and activity impairment (percentage activity loss). Higher percentages indicate worse outcomes.

### Statistical analyses

The ASAS criteria were used to classify patients as having nr-axSpA, AS or as not fulfilling the criteria (CLBP). Sociodemographic and clinical characteristics were summarized as mean and standard deviation (SD) or as median and interquartile range (IQR) and compared between subgroups using the unpaired *t* test or Wilcoxon rank sum test for continuous variables and the chi-square or Fisher exact test for categorical data.

Indirect standardization (by gender and age categories of 5 years) was used to calculate employment ratios for the total population and each disease subgroup (AS, nr-axSpA and CLBP), in comparison to the general Dutch population. Employment data from the general Dutch population was provided by the Dutch Centraal Bureau voor Statistiek (CBS) [21]. Poisson 95% confidence intervals (CI) for standardized proportions were calculated.

Scores in the WPAI are presented first as the proportion of patients with any (>0%) absenteeism, presenteeism, work or activity impairment and next as the average percentage absenteeism, presenteeism and work and activity impairment. Absenteeism, presenteeism and activity impairment were calculated only in employed patients, activity impairment in all patients. Differences between subgroups in the proportions of patients who had any restriction were tested using the chi-square and Fisher exact test. Differences between subgroups in the level of restriction in each subdomain of the WPAI were tested using the Wilcoxon rank sum test.

To investigate which factors are associated with each of the four domains of worker productivity, zero inflated negative binomial (ZINB) models were used. Zero inflated models are needed to adjust for the excess zeros in productivity outcomes (percentage zeros: absenteeism 87%; presenteeism 50%; work impairment 39%; activity impairment 32% of all observations). ZINB models assume that the zeros can result from two different processes; [22] the “certain zeros” (or always zeros) which are accounted for in the zero inflated logistic part and the “possible zeros” that are accounted for in the count part. As in the count part the values are over dispersed (i.e. the variance was much larger than the mean), the negative binomial distribution was preferred, and to fit the four subdomains of the WPAI, zero-inflated models were used [23].

To create the multivariable ZINB models four different steps were taken. In step 1 gender and age were included in both the binomial and count part of the ZINB model. In the second step all the candidate covariates (disease: CLBP, nr-axSpA or AS; education level: low, intermediate, high; occupation: manual vs. non-manual; duration of LBP (years); VAS score for pain; CRP, RMDQ score, ASDAS-CRP score and BASDAI score) were tested in univariate analysis in both the binomial and count part of the ZINB. All variables that were significant at *p* < 0.20 were considered for multivariable analyses in step 3. However, ASDAS-CRP and BASDAI were not validated in patients with CLBP, and as moderate correlation was seen between the VAS score for pain and the BASDAI score it was decided to take the ASDAS-CRP and BASDAI scores out of the multivariable model. In step 4, the model was repeated with the covariates with *p* values <0.05 in the multivariable analysis.

A ZINB provides regression coefficients for both the logistic and the count part separately. A positive coefficient in the zero inflated (logistic) part of the ZINB means that an increase in that variable leads to a higher likelihood of resulting in a “certain zero”’. A negative coefficient in the count part of the ZINB means that an increase of that variable leads to a smaller chance of scoring a zero in the outcome (i.e. subdomain of the WPAI).

The analyses were performed using STATA version 13.0 software (Stata Corporation TX, USA).

## Results

### CaFaSpA cohort

The enrollment of patients in the CaFaSpA 2 study has previously been described [14]. Overall, 2597 patients (ages 18–45 years) with CLBP from 38 primary care practices were invited to participate: 1161 patients (45%) responded to the invitation, among whom 480 expressed no interest in participation and 102 did not fulfil the inclusion criteria. In total there were 579 patients with CLBP included in this study. The median duration of LBP was 7 years (IQR 3–15 years), 41% of the patients were male and the mean age was 36.0 years (SD 7.0) (Table 1). In total 95 patients (16.4%) were classified as having axSpA, and 24 of those (25%) fulfilled the classification criteria for AS and 71 (75%) for nr-axSpA. The majority (59 out of 71) of the patients in the nr-axSpA group was classified based on MRI abnormalities. The percentage of women in the AS group was higher (75%) compared to the nr-axSpA (58%) and CLBP group (58%), although this difference was not statistically significant (*p* = 0.10). Of the three disease subgroups, patients with AS (8%) were less highly educated compared to patients with CLBP (21%) or nr-axSpA (24%). The percentage of patients with a manual occupation in the nr-axSpA group was 46% compared to 29% in the AS group and 37% in the patients with CLBP.

**Table 1:** Demographics and clinical characteristics in study participants (*n* = 579)

	CLBP (*n* = 484)	Nr-axSpA (*n* = 71)	AS (*n* = 24)
Age, mean (SD) years^a^	35.6 (7.1)	36.8 (6.6)	38.6 (5.8)
Male sex, *n* (%)	202 (42)	30 (42)	6 (25)
LBP duration, mean (SD) years	9.2 (7.7)	9.6 (7.4)	9.3 (9.9)
Disease activity			
VAS pain, median (IQR)^b^	5 (3–7)	4 (2–5)	4.5 (2–7)
BASDAI, median (IQR)	4.2 (2.3–6)	3.9 (2.4–5.4)	5.3 (2.9–6.6)
ASDAS-CRP, median (IQR)^c^	2.3 (1.6–2.9)	2.3 (1.6–2.9)	2.8 (2.1–3.5)
RMDQ, median (IQR)^d^	7 (3–13)	6 (3–9)	12 (5–17)
Educational level^e^			
Low (elementary school) (%)	177 (38)	29 (41)	11 (46)
Medium (high school) (%)	194 (41)	24 (34)	11 (46)
High (university) (%)	101 (21)	17 (24)	2 (8)
Work status			
Employed, *n* (%)	342 (72.2)	55 (77.5)	15 (62.5)
Disability pension, *n* (%)	8 (1.7)	2 (2.8)	0 (0)
Number of hours working per week, mean (SD)^f^	33.1 (9.4)	34.6 (8.6)	27.9 (12.4)
Occupation in employed patients^f^			
Manual, *n* (%)	124 (37)	24 (46)	4 (29)
Non-manual, *n* (%)	208 (63)	28 (54)	10 (71)

*CLBP* chronic low back pain, *Nr-axSpA* non-radiographic axial spondyloarthritis, *AS* ankylosing spondylitis, *IQR* interquartile range, *LBP* low back pain, *VAS* visual analog scale, *BASDA*I Bath Ankylosing Spondylitis Disease Activity Index, *ASDAS-CRP* Ankylosing Spondylitis Disease Activity Score-C-reactive protein, *RMDQ* Rolland Morris Disability Questionnaire. ^a^ *p* = 0.04 for CLBP vs. AS; ^b^ *p* = 0.04 for CLBP vs. nr-axSpA; ^c^ *p* = 0.01 for CLBP vs. AS; ^d^ *p* = 0.03 for CLBP vs. AS; ^e^total number of questionnaires about educational level: CLBP, *n* = 472 (12 missing), nr-axSpA, *n* = 70 (1 missing); ^f^total number of questionnaires about occupation and working hours in employed patients: CLBP, *n* = 332 (10 missing), AS, *n* = 14 (1 missing), nr-axSpA, n = 52 (3 missing)

### Work status

In total 342 out of 579 participants (72.4%) were employed. After adjusting for age, the likelihood of being employed was 0.92 (95% CI 0.86–0.99) and 0.88 (95%CI 0.81–0.94) for men and women, respectively, compared with the Dutch general population. Age-adjusted ratios for being employed, in patients with CLBP, nr-axSpA or AS were 0.89 (95% CI 0.84–0.94), 0.97 (95% CI 0.85–1.09) and 0.81 (95% CI 0.56–1.06), respectively. There were no patients with a disability pension in the newly identified AS group, while there were eight patients (1.7%) in the CLBP group and two patients (2.8%) in the newly identified nr-axSpA group.

### WPAI questionnaire

Of the 342 employed patients with CLBP, 318 (93%) completed the WPAI questionnaire, compared to 14/15 patients with AS (93%) and 48/55 patients with nr-axSpA (87%). Of the employed patients with AS, 14% had been absent from work in the past 7 days, while this percentage was 10% and 12% in the employed patients with nr-axSpA and with CLBP, respectively (Table 2). Presenteeism was the most prevalent in patients with CLBP (59%), but the percentage presenteeism was the highest in the AS group at 59%. There were no significant differences in any of the four sub scores between patients with CLBP and patients with nr-axSpA, or between patients with CLBP and patients with AS.

**Table 2:** Worker productivity assessed by the WPAI for employed patients with CLBP, nr-axSpA or AS (18–45 years of age)

	CLBP (*n* = 318)	Nr-axSpA (*n* = 48)	AS (*n* = 14)
Absenteeism			
Absenteeism present, *n* (% (95% CI))	38 (12 (8–16))	5 (10 (4–23))	2 (14 (2–43))
Absenteeism, mean % (SD)	53 (31)	47 (43)	54 (59)
Presenteeism			
Presenteeism present, *n* (% (95% CI))	188 (59 (53–64))	23 (47 (34–61))	7 (53 (27–79))
Presenteeism, mean % (SD)	45 (28)	46 (32)	59 (34)
Work impairment			
Work impairment present, *n* (% (95% CI))	197 (62 (56–67))	25 (52 (37–67))	8 (57 (29–82))
Work impairment, mean % (SD)	49 (30)	48 (33)	62 (36)
Activity impairment^a^			
Activity impairment present, *n* (% (95% CI))	322 (68 (63–72))	45 (63 (51–75))	19 (79 (58–93))
Activity impairment, mean % (SD)	51 (27)	49 (28)	56 (34)

*WPAI* Worker Productivity and Activity Impairment Questionnaire, *CLBP* chronic low back pain, *nr-axSpA* non-radiographic axial spondyloarthritis, *AS* ankylosing spondylitis. There were no significant differences in any of the four sub scores between patients with CLBP and patients with nr-axSpA, or between patients with CLBP and patients with AS. ^a^Calculated in all patients, not only in the employed patients (CLBP, *n* = 474; nr-axSpA, *n* = 71; AS, *n* = 24)

### ZINB models

Detailed results on the age-adjusted and gender-adjusted univariable regression can be found in Additional file 1, while the final multivariable model is presented in Table 3. In the final model the VAS score for pain and the RMDQ score were independently associated with the logistic part of each of the four domains of the WPAI. For the count part of the model, the VAS score for pain and the RMDQ score were independently associated with presenteeism, and work and activity impairment. This means that patients with pain and functional limitations are unlikely to have no restrictions in worker productivity (so unlikely to belong to the zero inflated part) and that increased pain and functional limitations are associated with more presenteeism, overall work impairment and activity impairment, but no absenteeism. In addition, lower educational level was associated with a likelihood of work impairment and level of overall work impairment, and with the level of presenteeism, and longer disease duration was associated with a decreased likelihood of work impairment.

**Table 3:** Final results of the ZINB regression model testing associations between demographical and clinical parameters and the WPAI subdomains corrected for age and gender

	Absenteeism		Presenteeism		Work impairment		Activity impairment	
Parameter	Count	Logistic	Count	Logistic	Count	Logistic	Count	Logistic
Education level								
Intermediate			0.100 (0.195)		-0.051 (0.578)	0.844 (0.004)		
High			-0.228 (0.015)		-0.252 (0.017)	0.716 (0.029)		
Duration of LBP (years)						0.043 (0.012)		
VAS pain		-0.190 (0.008)	0.081 (0.000)	-0.165 (<0.001)	0.079 (<0.001)	-0.324 (<0.001)	0.079 (<0.001)	-0.261 (<0.001)
RMDQ		-0.188 (<0.001)	0.048 (<0.001)	-0.005 (0.744)	0.058 (<0.001)	-0.152 (<0.001)	0.044 (<0.001)	-0.106 (<0.001)

Only the significant regression coefficients are shown, with the *p* values in parentheses. The logistic part of the ZINB model is generated for the “certain zero” cases, predicting whether or not a patient would be in this group. At the same time the count part of the model is predicting the counts for those patients who are not certain zeros.

*ZINB* zero inflated negative binomial, *WPAI* Worker Productivity and Activity Impairment Questionnaire, *LB*P low back pain, *VAS* = visual analog scale, *RDMQ* Roland Morris Disability Questionnaire

As an example of the interpretation of the output of the ZINB model; for activity impairment the “inflated” (logit) model predicting the “certain zeros” indicates that if a patient was to increase their VAS pain score by one point, the odds that the patient will belong in the “certain zero” group (have no activity impairment) would be a factor of exp(-0.261) = 0.770. In other words, the higher a patient’s VAS score the less likely the patient is to be a certain zero (have no activity impairment). On the other hand, the “count” part indicates that one point increase in the VAS pain score would increase activity impairment by a factor exp(0.079) = 1.082. Thus, the higher a patient’s VAS score, the more activity impairment is present.

## Discussion

To our knowledge this is the first study investigating the impact of yet undiagnosed nr-axSpA and AS on work outcomes within a group of patients with long-standing CLBP. The employment rate among our patients with CLBP was, as anticipated, lower than expected compared to the Dutch population of the same age and gender, and the lower employment rate was more pronounced in the patients with AS, although this was not significantly different. In addition the patients with AS reported the highest values of absenteeism, presenteeism and work and activity impairment, although the differences between patients with CLBP and nr-axSpA were not significant, perhaps due to the small number of patients with AS in the study population. Pain and functional limitations were associated with higher likelihood of having any work impairment. Pain by itself was associated with level of presenteeism, overall work impairment and activity impairment.

A comprehensive comparison with the existing literature is difficult as this is the first study addressing patients with previously unrecognized axSpA. Further, there is only limited literature on work outcomes in patients with nr-axSpA [24], and last but not least, data on employment and sick leave are country-specific, as the socioeconomic environment plays an important role [25]. Notwithstanding, a comparison that can be made involves a recent study in Dutch patients with early axSpA (defined based on the ESSG criteria), which evaluated problems in work participation. This study showed a remarkable high percentage of employed patients with axSpA, namely 81% [10]. This employment rate is even higher than the employment rate of the Dutch general population in 2014, which was 74.8% in the age category of 15–45 years [21]. An explanation of this high employment rate can be the high percentage of male participants in this study, which was 69% or the relatively low median BASDAI score (3.0) that was reported by the participants.

Focusing on the variables that are associated with work productivity in patients with axSpA, they are reported equally within different studies and settings. Several studies reported that variables measuring pain, disease activity and physical functioning are associated with reduced work productivity [10, 26–29]. This association is not only found in axSpA but also in other rheumatic conditions such as rheumatoid arthritis [30].

The strength of this study is our study population, which provided an unique opportunity to investigate work outcomes in as yet undiagnosed patients with nr-axSpA and with AS and to compare the findings with those in patients with long-standing CLBP. Moreover, none of the patients with nr-axSpA or AS had been diagnosed or treated by a rheumatologist for their disease, so there was no treatment bias in assessing the impact of the disease on work outcomes. A further strength of this study is the use of the ZINB models to assess associations with work productivity; this is a very elegant statistical technique using a logistic and a count model at the same time, creating an optimal fit for our data with excessive zeros [23].

A limitation of our study is the cross-sectional design, which limits any conclusions about causality. The risk of sampling bias was minimized by using only simple inclusion criteria (age 18–45 years and CLBP lasting more than 3 months). We did not include coping in our analyses, therefore is it unclear to which extent coping plays a role in the self-reported questionnaires such as the VAS for pain and the RMDQ. Not all participants answered the questions on work outcomes; however, the response rate for those questionnaires was 92% and there were no difference in patients’ characteristics between the responders and non-responders. Another potential limitation is that it is unclear why the patients with nr-axSpA or AS have not been recognized by their primary care physicians and referred to a rheumatologist; is this because their primary care physician had limited knowledge about axSpA and did not recognize those patients or because the patients had so far experienced few symptoms from their disease? The second reason seems unlikely, as the median BASDAI score was 3.9 and 5.2 in patients with nr-axSpA and AS, respectively. Also, selection bias towards more patients with more severe CLBP seems unlikely, as the median VAS score in our study is comparable to or even lower than VAS scores in other cohorts with low back pain [31]. Finally, the group of patients with AS only contained 24 patients, of whom only 15 were employed, making it hard to find significant differences between the AS, nr-axSpA and CLBP groups.

On this line it should be noted that there was a high percentage women in our AS sample (75%); no other AS cohorts are reported to have such a high percentage. A possible explanation can be that we used a different approach to including patients in our study; we only selected based on age and the presence of CLBP. In other cohorts patients have been selected by predefined features specific to axSpA, leading to the possibility that male participants are easier to include. On the other hand, this finding could be an indication that at this moment more female patients with AS are being missed by primary care physicians, indicating an opportunity to educate primary care physicians more thoroughly about axSpA.

It is important to conduct research on work productivity, absenteeism and presenteeism in as yet undiagnosed patients with axSpA, as they can be indicators of future work disability [32]. Our results show that there are significant associations between patient-reported outcome measurement (PROMs) such as pain and functional limitations and work productivity, but no associations between more objective variables such as age, gender, disease (CLBP, nr-axSpA or AS), manual occupation and work productivity; this leads to the cautious conclusion that the impact on work productivity is not disease-related but related to the degree of pain and physical limitations a patients experiences. This finding is encouraging as we know from previous studies that adequately treating patients with AS or nr-axSpA leads to an improvement in PROMs [33]. Moreover, after starting treatment, not only is an improvement in work participation reported, but also an improvement in unpaid work is reported in patients with AS and with nr-axSpA [11, 34]. A recent study in southern Sweden in patients with nr-axSpA showed a sustained improvement in work disability even two years after the start of treatment [35]. These findings emphasize that is important and useful to recognize patients suspected of having axSpA early and refer them to a rheumatologist.

## Conclusions

Our findings demonstrate that the impact of as yet undiagnosed nr-axSpA and AS is substantial although the outcomes in work productivity were not significantly different from those in patients with long-standing CLBP. Variables associated with reduced work productivity were mainly PROMs such as VAS pain score and functional limitations measured by the RMDQ. Early recognition and subsequently adequate treatment of yet undiagnosed patients with nr-axSpA and with AS can potentially lead to maintaining optimal work productivity in patients with nr-axSpA and with AS and a reduction in indirect costs.

## Additional file

Additional file 1: Modeling steps by WPAI subdomain using zero inflated negative binomial (ZINB) regression models. Modeling steps to create per subdomain of the WPAI a zero inflated negative binomial (ZINB) regression model to explore associations between WPAI subdomains and demographical and clinical variables (DOCX 23 kb)

## Abbreviations

AS: Ankylosing spondylitis
ASAS: Assessment of SpondyloArthritis International Society
ASDAS: Ankylosing Spondylitis Disease Activity Score
axSpA: Axial spondyloarthritis
BASDAI: Bath Ankylosing Spondylitis Disease Activity Index
CaFaSpA: Case Finding Axial Spondyloarthrits
CBS: Centraal Bureau Statistiek
CI: Confidence interval
CLBP: Chronic low back pain
CRP: C-reactive protein
ESR: Erythrocyte sedimentation rate
GP: General Practitioner
HLA-B27: Human leukocyte antigen B27
ICPC: International Classification of Primary Care
ISCO: International Standard Classification of Occupation
MRI: Magnetic resonance imaging
Nr-axSpA: Non-radiographic axial spondyloarthritis
RMDQ: Rolland Morris Disability Questionnaire
SD: Standard deviation
VAS: Visual analogue scale
WPAI: Work Productivity and Activity Impairment
ZINB: Zero inflated negative binomial

## References

[1] Hoy D, Bain C, Williams G, March L, Brooks P, Blyth F, Woolf A, Vos T, Buchbinder R (2012). A systematic review of the global prevalence of low back pain. Arthritis Rheum.

[2] Freburger JK, Holmes GM, Agans RP, Jackman AM, Darter JD, Wallace AS, Castel LD, Kalsbeek WD, Carey TS (2009). The rising prevalence of chronic low back pain. Arch Intern Med.

[3] van Hoeven L, Luime J, Han H, Vergouwe Y, Weel A (2014). Identifying axial spondyloarthritis in Dutch primary care patients, ages 20-45 years, with chronic low back pain. Arthritis Care Res (Hoboken).

[4] Rudwaleit M, van der Heijde D, Landewe R, Listing J, Akkoc N, Brandt J, Braun J, Chou CT, Collantes-Estevez E, Dougados M (2009). The development of Assessment of SpondyloArthritis International Society classification criteria for axial spondyloarthritis (part II): validation and final selection. Ann Rheum Dis.

[5] Poddubnyy D, Brandt H, Vahldiek J, Spiller I, Song IH, Rudwaleit M, Sieper J (2012). The frequency of non-radiographic axial spondyloarthritis in relation to symptom duration in patients referred because of chronic back pain: results from the Berlin early spondyloarthritis clinic. Ann Rheum Dis.

[6] Kiltz U, Baraliakos X, Karakostas P, Igelmann M, Kalthoff L, Klink C, Krause D, Schmitz-Bortz E, Florecke M, Bollow M (2012). Do patients with non-radiographic axial spondylarthritis differ from patients with ankylosing spondylitis?. Arthritis Care Res (Hoboken).

[7] Kiltz U, van der Heijde D (2009). Health-related quality of life in patients with rheumatoid arthritis and in patients with ankylosing spondylitis. Clin Exp Rheumatol.

[8] Ramonda R, Marchesoni A, Carletto A, Bianchi G, Cutolo M, Ferraccioli G, Fusaro E, De Vita S, Galeazzi M, Gerli R (2016). Patient-reported impact of spondyloarthritis on work disability and working life: the ATLANTIS survey. Arthritis Res Ther.

[9] Boonen A, van der Linden SM (2006). The burden of ankylosing spondylitis. J Rheumatol Suppl.

[10] van der Weijden MA, Boonen A, van der Horst-Bruinsma IE (2014). Problems in work participation and resource use should not be underestimated in patients with early spondyloarthritis. J Rheumatol.

[11] van der Heijde D, Joshi A, Pangan AL, Chen N, Betts K, Mittal M, Bao Y (2015). ASAS40 and ASDAS clinical responses in the ABILITY-1 clinical trial translate to meaningful improvements in physical function, health-related quality of life and work productivity in patients with non-radiographic axial spondyloarthritis. Rheumatology (Oxford).

[12] Hoy D, March L, Brooks P, Blyth F, Woolf A, Bain C, Williams G, Smith E, Vos T, Barendregt J (2014). The global burden of low back pain: estimates from the Global Burden of Disease 2010 study. Ann Rheum Dis.

[13] Froud R, Patterson S, Eldridge S, Seale C, Pincus T, Rajendran D, Fossum C, Underwood M (2014). A systematic review and meta-synthesis of the impact of low back pain on people's lives. BMC Musculoskelet Disord.

[14] van Hoeven L, Vergouwe Y, de Buck PD, Luime JJ, Hazes JM, Weel AE (2015). External validation of a referral rule for axial spondyloarthritis in primary care patients with chronic low back pain. PLoS One.

[15] Sieper J, Rudwaleit M, Baraliakos X, Brandt J, Braun J, Burgos-Vargas R, Dougados M, Hermann KG, Landewe R, Maksymowych W (2009). The Assessment of SpondyloArthritis International Society (ASAS) handbook: a guide to assess spondyloarthritis. Ann Rheum Dis.

[16] Goie The HS, Steven MM, van der Linden SM, Cats A (1985). Evaluation of diagnostic criteria for ankylosing spondylitis: a comparison of the Rome, New York and modified New York criteria in patients with a positive clinical history screening test for ankylosing spondylitis. Br J Rheumatol.

[17] Zochling J (2011). Measures of symptoms and disease status in ankylosing spondylitis: Ankylosing Spondylitis Disease Activity Score (ASDAS), Ankylosing Spondylitis Quality of Life Scale (ASQoL), Bath Ankylosing Spondylitis Disease Activity Index (BASDAI), Bath Ankylosing Spondylitis Functional Index (BASFI), Bath Ankylosing Spondylitis Global Score (BAS-G), Bath Ankylosing Spondylitis Metrology Index (BASMI), Dougados Functional Index (DFI), and Health Assessment Questionnaire for the Spondylarthropathies (HAQ-S). Arthritis Care Res (Hoboken).

[18] Roland M, Morris R (1983). A study of the natural history of back pain. Part I: development of a reliable and sensitive measure of disability in low-back pain. Spine (Phila Pa 1976).

[19] ISCO-08: http://www.ilo.org/public/english/bureau/stat/isco/isco08/.

[20] Reilly MC, Gooch KL, Wong RL, Kupper H, van der Heijde D (2010). Validity, reliability and responsiveness of the Work Productivity and Activity Impairment Questionnaire in ankylosing spondylitis. Rheumatology (Oxford).

[21] CBS: http://statline.cbs.nl/Statweb/. 2014.

[22] Yau KKW, Wang K, Lee AH (2003). Zero-inflated negative binomial mixed regression modeling of over-dispersed count data with extra zeros. Biom J.

[23] Greene WH (1994). Accounting for excess zeros and sample selection in Poisson and negative binomial regression models.

[24] Boonen A, Sieper J, van der Heijde D, Dougados M, Bukowski JF, Valluri S, Vlahos B, Kotak S. The burden of non-radiographic axial spondyloarthritis. Semin Arthritis Rheum. 2015;44(5):556-62. 10.1016/j.semarthrit.2014.10.009 25532945

[25] Wynne-Jones G, Cowen J, Jordan JL, Uthman O, Main CJ, Glozier N, van der Windt D (2014). Absence from work and return to work in people with back pain: a systematic review and meta-analysis. Occup Environ Med.

[26] Haglund E, Bremander A, Bergman S, Jacobsson LT, Petersson IF (2013). Work productivity in a population-based cohort of patients with spondyloarthritis. Rheumatology (Oxford).

[27] Boonen A, Brinkhuizen T, Landewe R, van der Heijde D, Severens JL (2010). Impact of ankylosing spondylitis on sick leave, presenteeism and unpaid productivity, and estimation of the societal cost. Ann Rheum Dis.

[28] Boonen A, Chorus A, Miedema H, van der Heijde D, van der Tempel H, van der Linden S (2001). Employment, work disability, and work days lost in patients with ankylosing spondylitis: a cross sectional study of Dutch patients. Ann Rheum Dis.

[29] de Hooge M, Ramonda R, Lorenzin M, Frallonardo P, Punzi L, Ortolan A, Doria A (2016). Work productivity is associated with disease activity and functional ability in Italian patients with early axial spondyloarthritis: an observational study from the SPACE cohort. Arthritis Res Ther.

[30] Geuskens GA, Hazes JM, Barendregt PJ, Burdorf A (2008). Predictors of sick leave and reduced productivity at work among persons with early inflammatory joint conditions. Scand J Work Environ Health.

[31] Gordon A, Callaghan D, Spink D, Cloutier C, Dzongowski P, O'Mahony W, Sinclair D, Rashiq S, Buckley N, Cohen G (2010). Buprenorphine transdermal system in adults with chronic low back pain: a randomized, double-blind, placebo-controlled crossover study, followed by an open-label extension phase. Clin Ther.

[32] Mau W, Listing J, Huscher D, Zeidler H, Zink A (2005). Employment across chronic inflammatory rheumatic diseases and comparison with the general population. J Rheumatol.

[33] Callhoff J, Sieper J, Weiss A, Zink A, Listing J (2015). Efficacy of TNFalpha blockers in patients with ankylosing spondylitis and non-radiographic axial spondyloarthritis: a meta-analysis. Ann Rheum Dis.

[34] Barkham N, Coates LC, Keen H, Hensor E, Fraser A, Redmond A, Cawkwell L, Emery P (2010). Double-blind placebo-controlled trial of etanercept in the prevention of work disability in ankylosing spondylitis. Ann Rheum Dis.

[35] Wallman JK, Joud A, Olofsson T, Jacobsson LT, Bliddal H, Kristensen LE. Work disability in non-radiographic axial spondyloarthritis patients before and after start of anti-TNF therapy: a population-based regional cohort study from southern Sweden. Rheumatology (Oxford). 2017. 10.1093/rheumatology/kew473 28064208

