# Supplementary material for: Work outcome in yet undiagnosed patients with non-radiographic axial spondyloarthritis and ankylosing spondylitis; results of a cross-sectional study among patients with chronic low back pain
Source: Arthritis Res Ther. 2017 Jun 17;19:143. doi: 10.1186/s13075-017-1333-x (PMC5474026; doi:10.1186/s13075-017-1333-x)
Supplement: Additional file 1: — Modeling steps by WPAI subdomain using zero inflated negative binomial (ZINB) regression models. Modeling steps to create per subdomain of the WPAI a zero inflated negative binomial (ZINB) regression model to explore associations between WPAI subdomains and demographical and clinical variables (DOCX 23 kb) [file 13075_2017_1333_MOESM1_ESM.docx]

**Additional file by article** ‘Work-outcome in yet undiagnosed patients with non-radiographic axial spondyloarthritis and ankylosing spondylitis; results of a cross-sectional study among patients with chronic low back pain’

| **Table 1.**  **Modelling steps by WPAI subdomain using zero inflated negative binomial (ZINB) regression models *** | | | | | | | | |
| --- | --- | --- | --- | --- | --- | --- | --- | --- |
| *Step 2: Univariate analyses with candidate covariates corrected for gender and age. Covariates with p <0.20 are maintained in the multivariate model (step 3)* | | | | | | | | |
|  | **Absenteeism** | | **Presenteeism** | | **Work Impairment** | | **Activity impairment** | |
| **Variable** | Binomial | Logit | Binomial | Logit | Binomial | Logit | Binomial | Logit |
| Disease‡ |  |  |  |  |  |  |  |  |
| Nr-axSpA | -0.366 (0.313 | 0.254 (0.612) | -0.021 (0.869) | **0.341 (0.186)** | -0.012 (0.934) | **0.407 (0.194)** | -0.034 (0.725) | 0.221 (0.411) |
| AS | -0.113 (0.826) | -0.093 (0.905) | **0.281 (0.112)** | -0.448 (0.303) | 0.248 (0.324) | 0.229 (0.682) | 0.086 (0.554) | - 0.441 (0.396) |
| Education level§ |  |  |  |  |  |  |  |  |
| Intermediate | -0.195 (0.438) | **0.927 (0.009)** | **-0.180 (0.043)** | 0.095 (0.620) | -0.118 (0.263) | **0.839 (0.001)** | **-0.139 (0.049)** | **0.494 (0.020)** |
| High | -0.243 (0.392) | **0.873 (0.036)** | **-0.361 (0.001)** | 0.285 (0.213) | **-0.363 (0.003)** | **0.884 (0.003)** | **-0.142 (0.098)** | **0.635 (0.010)** |
| Manueel occupation‡ | 0.205 (0.372) | **0.904 (0.006)** | **-0.173 (0.062)** | **0.509 (0.019)** | **-0.156 (0.105)** | **0.661 (0.004)** | -0.067 (0.414) | 0.134 (0.538) |
| Duration LBP | 0.005 (0.686) | **0.041 (0.081)** | 0.005 (0.367) | 0.015 (0.205) | 0.000 (0.901) | **0.023 (0.113)** | -0.000 (0.828) | **0.023 (0.067)** |
| Vas pain | -0.003 (0.949) | -**0.305 (<0.001)** | **0.116 (<0.001)** | **-0.171 (<0.001)** | **0.105 (<0.001)** | **-0.338 (<0.001)** | **0.116 (<0.001)** | **-0.317 (<0.001)** |
| ASDAS-CRP | -0.136 (0.399) | **-1.056 (<0.001)** | **0.292 (<0.001)** | **-0.507 (<0.001)** | **0.269 (<0.001)** | **-.0898 (<0.001)** | **0.304 (<0.001)** | **-0.746 (<0.001)** |
| BASDAI | -0.290 (0.582) | **-0.375 (<0.001)** | **0.117 (<0.001)** | **-0.164 (<0.001)** | **0.113 (<0.001)** | **-0.342 (<0.001)** | **0.111 (<0.001)** | **-0.354 (<0.001)** |
| RMDQ | **0.036 (0.094)** | **-0.204 (<0.001)** | **0.059 (<0.001)** | **-0.031 (0.029)** | **0.066 (<0.001)** | **-0.124 (<0.001)** | **0.056 (<0.001)** | **-0.137 (<0.001)** |
| CRP | -0.012 (0.660) | -0.010 (0.770) | 0.004 (0.635) | **-0.046 (0.036)** | -0.001 (0.933) | -0.026 (0.323) | -0.000 (0.983) | 0.022 (0.296) |
| *The estimated regression coefficients are given, the p-value is between the brackets. The logit model of the ZINB is generated for the ‘certain zero’ cases, predicting whether or not a patient would be in this group. At the same time a negative binomial model is generated predicting the counts for those patients who are not certain zeros.  ‡ CLBP as reference, nr-axSpA coded as 1, AS coded as 2; §Low education level as reference, intermediate level coded as 1, high level coded as 2; LBP= low back pain; VAS = visual analogue scale; ASDAS-CRP = Ankylosing Spondylitis Disease Activity Score with c-reactive protein; BASDAI = Bath Ankylosing Spondylitis Disease Activity Index; RDMQ = Roland Morris Disability Questionnaire; CRP = C-reactive protein | | | | | | | | |

| *Step 3. Multivariate analyses with significant and applicable covariates corrected for gender and age.† Covariates with p<0.05 are maintained for step 4* | | | | | | | | |
| --- | --- | --- | --- | --- | --- | --- | --- | --- |
|  | **Absenteeism** | | **Presenteeism** | | **Work Impairment** | | **Activity impairment** | |
| **Variable** | Binomial | Logit | Binomial | Logit | Binomial | Logit | Binomial | Logit |
| Disease‡ |  |  |  |  |  |  |  |  |
| Nr-axspa |  |  | 0.179 (0.155) | 0.239 (0.467) |  | 0.156 (0.663) |  |  |
| AS |  |  | 0.012 (0.956) | 0.231 (0.711) |  | 0.216 (0.747) |  |  |
| Education level§ |  |  |  |  |  |  |  |  |
| Intermediate |  | 0.606 (0.144) | -0.082 (0.371) |  | -0.062 (0.505) | **0.820 (0.007)** | -0.051 (0.381) | 0.231 (0.326) |
| High |  | 0.309 (0.563) | **-0.223 (0.047)** |  | -**0.290 (0.011)** | 0.489 (0.164) | -0.067 (0.337) | 0.303 (0.269) |
| Manueel occupation |  | 0.571 (0.152) | 0.020 (0.819) | 0.404 (0.095) | 0.053 (0.541) | 0.537 (0.051) |  |  |
| Duration LBP |  | 0.053 (0.057) |  |  |  | **0.047 (0.008)** |  |  |
| Vas pain |  | **-0.219 (0.004)** | **0.095 (<0.001)** | **-0.323 (<0.001)** | **0.082 (<0.001)** | **-0.340 (<0.001)** | **0.080 (<0.001)** | **-0.267 (<0.001)** |
| RMDQ | 0.044 (0.054) | **-0.172 (<0.001)** | **0.052 (<0.001)** | **-0.092 (0.003)** | **0.058 (<0.001)** | **-0.090 (<0.001)** | **0.044 (<0.001)** | **-0.104 (<0.001)** |
| CRP |  |  |  | **-**0.057 (0.072) |  |  |  |  |
| *The estimated regression coefficients are given, the p-value is between the brackets. The logit model of the ZINB is generated for the ‘certain zero’ cases, predicting whether or not a patient would be in this group. At the same time a negative binomial model is generated predicting the counts for those patients who are not certain zeros.  † BASDAI and ASDAS-CRP were excluded after step 2 as both are not validated to use in CLBP patients.  ‡ CLBP as reference, nr-axSpA coded as 1, AS coded as 2; §Low education level as reference, intermediate level coded as 1, high level coded as 2; LBP = low back pian; VAS = visual analogue scale; RDMQ = Roland Morris Disability Questionnaire; CRP = C-reactive protein | | | | | | | | |
